# Clinically Significant ISUP Upgrading in the Multiparametric MRI Era: Biopsy Tumor Burden Outperforms Complex Machine Learning Models in a Single-Center Exploratory Cohort

**DOI:** 10.3390/cancers18050730

**Published:** 2026-02-24

**Authors:** Cristian Condoiu, Adelina Baloi, Dorel Sandesc, Alin Adrian Cumpanas, Silviu Latcu, Vlad Dema, Radu Caprariu, Alina Cristina Barb, Andreea Ciucurita, Adelina Marinescu, Talida Georgiana Cut, Razvan Bardan

**Affiliations:** 1 Doctoral School, Victor Babes University of Medicine and Pharmacy Timisoara, E. Murgu Square, No. 2, 300041 Timisoara, Romania; cristian.condoiu@umft.ro (C.C.); silviu.latcu@umft.ro (S.L.); vlad.dema@umft.ro (V.D.); andreea.ciucurita@umft.ro (A.C.); 2 Department X—Surgery II, Discipline Anesthesia and Intensive Care, Victor Babes University of Medicine and Pharmacy Timisoara, E. Murgu Square, Nr. 2, 300041 Timisoara, Romania; sandesc.dorel@umft.ro; 3 Department XV, Discipline of Urology, Victor Babes University of Medicine and Pharmacy Timisoara, E. Murgu Square, No. 2, 300041 Timisoara, Romania; cumpanas.alin@umft.ro (A.A.C.); razvan.bardan@umft.ro (R.B.); 4 Department XV, Discipline of Radiology and Medical Imaging, University of Medicine and Pharmacy ‘’Victor Babes’’ Timisoara, E. Murgu Square, Nr. 2, 300041 Timisoara, Romania; 5 Department II of Microscopic Morphology, Victor Babes University of Medicine and Pharmacy Timisoara, E. Murgu Square, No. 2, 300041 Timisoara, Romania; toma.alina@umft.ro; 6 Department XIII, Discipline of Infectious Diseases, Victor Babes University of Medicine and Pharmacy Timisoara, E. Murgu Square, No. 2, 300041 Timisoara, Romania; adelina.marinescu@umft.ro (A.M.); talida.cut@umft.ro (T.G.C.)

**Keywords:** prostate cancer, precision medicine, artificial intelligence, personalized treatment, imaging modalities, risk stratification, ISUP upgrading, PSA density, positive core ratio, multiparametric MRI

## Abstract

**Background/Objectives**: Despite multiparametric MRI (mpMRI)-guided biopsy, clinically significant upgrading (CSU) of ISUP Grade Group (GG) at radical prostatectomy (RP) remains common in prostate cancer (PCa). We aimed to identify predictors of CSU (biopsy GG ≤ 2 to RP GG ≥ 3) using routine preoperative variables, and to benchmark a parsimonious logistic model against multiple machine learning (ML) classifiers. **Methods**: In this single-center exploratory analysis, 96 consecutive PCa patients underwent pre-biopsy mpMRI, systematic ± MRI-targeted biopsy, and RP. Predictive modeling was restricted to biopsy GG 1–2 patients (*n* = 64). LASSO-guided feature selection and Firth-penalized logistic regression were used to build a locked reference model, evaluated against ML classifiers using cross-validated discrimination, calibration, and decision curve analysis. **Results**: CSU occurred in 10/64 patients (15.6%). Positive core ratio was the dominant independent predictor (adjusted OR 1.54 per 10% increase, 95% CI 1.10–2.17). PSA density (PSAD) showed a consistent positive association but did not retain independent significance. The locked two-variable model (AUC ≈ 0.75–0.79) outperformed all ML classifiers in discrimination, calibration, and net clinical benefit; however, the limited event count (n = 10) constrains model stability, and these findings require external validation. **Conclusions**: In a PCa mpMRI-informed diagnostic pathway, CSU is primarily driven by biopsy tumor burden. A simple logistic model based on positive core ratio and PSAD outperformed more complex ML approaches in this exploratory cohort, supporting integration of biopsy tumor burden metrics into preoperative risk stratification pending external validation.

## Simple Summary

Sometimes, a prostate biopsy underestimates how aggressive the cancer is. This study looked at ways to predict when the final surgery will find a higher-grade cancer than the initial biopsy. We analyzed 96 men who had both a biopsy and their prostate removed. We focused on factors like PSA (a blood test for prostate cancer), MRI scans, and biopsy results. We found that the extent of cancer involvement in biopsy cores, and to a lesser degree PSA density, were associated with upgrading risk to a more aggressive cancer at surgery. Using just these two factors, a simple statistical model predicted grade upgrading more accurately than more complex computer models. If confirmed in larger studies, this tool could help doctors identify patients who have more aggressive cancer than initially thought, so they can choose the best treatment.

## 1. Introduction

Prostate cancer (PCa) remains one of the most common malignancies in men worldwide, with ~1.4 million new cases diagnosed annually [1,2]. Diagnosis typically involves a combination of prostate-specific antigen (PSA) testing and digital rectal examination (DRE), followed by imaging and biopsy for those with clinical suspicion. The International Society of Urological Pathology (ISUP) Grade Group (GG) system [3,4] now underpins clinical risk stratification. However, ISUP GG migration between biopsy and radical prostatectomy (RP) remains a major challenge: roughly 25–40% of patients are upgraded at surgery [5,6], with only 40–60% demonstrating concordant grading [7,8]. This grade discordance has direct clinical consequences, as patients initially classified as low-grade (usually considered appropriate for active surveillance [9]), who actually harbor occult higher-grade disease risk undertreatment, whereas grade overestimation may lead to unnecessary intervention. In fact, upgrading is also seemingly associated with adverse pathological features (such as extra-prostatic extension), positive surgical margins, and higher rates of biochemical recurrence [7,10].

Improving the accuracy of initial grading is therefore crucial. Multiparametric magnetic resonance imaging (mpMRI) of the prostate has become a standard adjunct before biopsy, as it can identify suspicious lesions and guide targeted sampling [11,12,13]. The PROMIS trial, for example, demonstrated 18% more clinically significant cancers (GG ≥ 2) detected when using mpMRI-targeted sampling, as opposed to the traditional 12-core transrectal ultrasound (TRUS)-guided biopsy [14]. Current guidelines recommend pre-biopsy mpMRI and combined (systematic plus targeted) prostate tissue sampling in biopsy-naive patients [15,16]. Even so, while this combined approach improves detection of clinically significant PCa [14], ISUP upgrading at RP still persists: a recent meta-analysis found that ~30% of men were upgraded at surgery, despite having undergone MRI-targeted biopsies [17]. Therefore, while mpMRI improves detection of aggressive disease, it does not reliably eliminate under-sampling of high-grade components, underscoring persistent limitations in preoperative PCa characterization.

Several factors contribute to these discrepancies. Prostate tumors are often multifocal and heterogeneous [18], and systematic biopsy samples only a small fraction of the gland (<1% of prostate volume [19]), increasing the risk of missing clinically significant disease. Even with image guidance, mpMRI may underestimate disease extent, as lesion size does not always correlate with pathological aggressiveness [20]. Interobserver variability in both radiologic interpretation and histopathological grading further compounds this uncertainty [21,22,23].

Given the risk of occult higher-grade PCa, numerous studies have explored preoperative predictors of upgrading. PSA density (PSAD), defined as serum PSA divided by prostate volume, is consistently associated with upgrading risk, with proposed thresholds ranging from 0.15–0.25 ng/mL^2^ [24,25,26,27]. Biopsy tumor burden metrics—mainly the number/percentage of positive cores and the maximum cancer length in a core—also correlate with upgrading risk [7], as greater histologic involvement likely reflects more extensive disease not fully captured by grade alone. The presence of perineural invasion (PNI) on biopsy has additionally been associated with upgrading in selected cohorts [28,29].

Imaging features on mpMRI further refine risk stratification in urologic malignancies [30,31,32] and help prevent unnecessary treatment-associated morbidity [33,34,35]. Higher PI-RADS scores and MRI-visible lesions are more frequently observed in patients who demonstrate upgrading [36]. PI-RADS 4–5 lesions, in particular, are associated with increased upgrading risk at RP [37]. Incorporation of MRI-derived variables into predictive models has been shown to improve discrimination, with reported concordance indices exceeding those of biopsy-only models [38]. Radiologic signs of extra-prostatic extension or larger lesion volume may further indicate biologically aggressive disease.

Multiple pre-treatment risk stratification tools are used in clinical practice to guide management, including the traditional D’Amico classification [39], the National Comprehensive Cancer Network (NCCN) risk categories [40], the University of California, San Francisco–Cancer of the Prostate Risk Assessment (UCSF-CAPRA) score [41], and the more recent Cambridge Prognostic Group (CPG) classification system [42]. While these instruments are valuable for estimating recurrence and progression risk, they implicitly assume accurate biopsy grading. In the presence of ISUP GG migration, patients may be systematically misclassified, most often into lower-risk categories, which in turn directly influences treatment selection, surgical planning, and eligibility for active surveillance.

Recognizing these limitations, there is growing interest in predictive models specifically targeting ISUP GG migration. Multivariable nomograms integrating clinical, imaging, and biopsy parameters have demonstrated improved predictive performance [38]. More recently, machine learning (ML) approaches have been explored to capture complex, non-linear relationships among predictors. While several studies have reported promising discrimination [38,43,44], many ML models (MLMs) suffer from overfitting, limited external validation, and reduced interpretability [45,46]. Accordingly, their role in routine clinical practice remains investigational.

Given these considerations, the present study aimed to evaluate whether routinely available preoperative variables—including PSA-derived metrics, mpMRI features, and detailed biopsy histopathological parameters—can reliably predict clinically significant upgrading (CSU), defined as an increase from biopsy ISUP GG ≤ 2 to post-RP GG ≥ 3, in a cohort of biopsy-diagnosed PCa patients undergoing definitive surgical treatment. By identifying predictors of such ISUP GG migration, we seek to improve preoperative risk stratification and support better individualized treatment planning.

## 2. Materials and Methods

### 2.1. Study Design

This study represents a retrospective, single-center cohort analysis of consecutive patients diagnosed with PCa who underwent pre-biopsy mpMRI as part of their initial work-up, to be then confirmed via systematic ± MRI-targeted TRUS-guided biopsy and finally undergo open retropubic RP within the Department of Urology, Pius Brinzeu County Hospital, Timișoara, Romania. The study period spanned January 2023 to December 2025. All patients undergoing RP during this interval in our center were retrospectively screened and reviewed for eligibility, according to the relevant procedural codes, then independently verified. The study protocol was approved by the institutional ethics committee (72/2 October 2023), and the analysis was conducted using fully de-identified data. Informed consent for diagnostic and therapeutic procedures, as well as for retrospective analysis of anonymized data, was obtained from all patients at hospital admission, in accordance with institutional practice and the Declaration of Helsinki.

Patients were included if they met all of the following criteria: (1) histologically confirmed PCa (acinar adenocarcinoma/variant histology) on TRUS-guided biopsy; (2) availability of pre-biopsy mpMRI, performed according to institutional protocol; (3) underwent systematic ± MRI-targeted prostate biopsy, followed by RP within our institution; (4) complete clinical, imaging, biopsy, and final pathological data available for analysis.

Conversely, patients were excluded if they met any of the following: (1) unavailable or incomplete biopsy pathology reports; (2) prior PCa-directed therapy, including androgen deprivation therapy, radiotherapy, chemotherapy, or focal therapy; (3) prostatectomy performed as part of radical and/or salvage cystectomy for bladder cancer, perforation or pelvic abscess.

During the three-year study period, 96 consecutive patients fulfilled all inclusion criteria and constituted the final analytical population.

### 2.2. Data Collection

#### 2.2.1. Clinical and Laboratory Parameters

Baseline clinical and laboratory data were extracted retrospectively from electronic medical records using standardized data collection templates to ensure consistency.

Collected variables included age at diagnosis (years); pre-biopsy serum PSA (ng/mL), measured within 30 days prior to biopsy; TRUS-measured prostate volume (mL); calculated PSAD (PSA/prostate volume, ng/mL^2^); and DRE findings, classified as negative (normal) or positive (suspicious for malignancy). Clinical staging (cT and cN categories) was assigned using a combination of DRE findings and mpMRI results, in accordance with contemporary TNM staging guidelines.

All patients included in the final analytical cohort had complete data for all clinical, imaging, and biopsy variables included in the analysis. Patients with unavailable or incomplete biopsy pathology reports were excluded a priori (see exclusion criteria). No imputation was required, as the study cohort was restricted to consecutive patients with complete preoperative datasets. This complete-case design, while ensuring analytical consistency, may introduce selection bias if data missingness was non-random.

#### 2.2.2. Multiparametric MRI Acquisition Protocol and Interpretation

All patients underwent pre-biopsy mpMRI as part of standard diagnostic evaluation. MRI examinations were performed on 1.5 Tesla or 3.0 Tesla scanners, depending on clinical availability. Scanner field strength was not systematically recorded in the study database; therefore, stratified analysis by field strength or formal harmonization of MRI-derived variables across scanners was not feasible. The standardized acquisition protocol included: (1) High-resolution axial, sagittal, and coronal T2-weighted imaging. (2) Diffusion-weighted imaging (DWI) with multiple b-values (0, 100, 800, and ≥1500 s/mm^2^). (3) Apparent diffusion coefficient (ADC) map reconstruction. (4) Dynamic contrast-enhanced (DCE) imaging following intravenous administration of gadolinium-based contrast agent (0.1 mmol/kg), with temporal resolution below 10 s.

All MRI studies were interpreted by experienced radiologists using PI-RADS version 2.1 criteria, with discrepancies being resolved by consensus. For each examination, the overall PI-RADS score of the index lesion, number of suspicious lesions, lesion laterality (unilateral vs. bilateral), maximum lesion diameter (mm), and the presence of MRI-suspected extracapsular extension (ECE), seminal vesicle invasion (SVI), or suspicious pelvic lymphadenopathy were prospectively recorded.

#### 2.2.3. Biopsy Technique and Histopathological Assessment

All patients underwent TRUS-guided prostate biopsy under local anesthesia with periprostatic nerve block. Biopsies were performed using an 18-gauge automated biopsy needle. The standard biopsy protocol consisted of 12 systematic cores, sampling the lateral and medial aspects of the base, mid-gland, and apex bilaterally. In patients with MRI-visible lesions (PI-RADS ≥ 3), additional targeted cores (typically 2–4 per lesion, with a maximum of 6 additional cores) were obtained using a cognitive fusion technique, reflecting routine clinical practice.

All TRUS-guided biopsies, including cognitive fusion targeting, were performed by a small group of experienced urologists within a single department. Although individual operator identifiers were not recorded, the limited number of operators and adherence to a standardized biopsy protocol were intended to reduce procedural variability.

Biopsy specimens were evaluated by dedicated genitourinary pathologists according to current ISUP recommendations. Gleason grading and ISUP grade group assignment were performed independently, with consensus review for borderline or discordant cases.

Recorded biopsy parameters included primary and secondary Gleason patterns; ISUP grade group (GG = 1–5); total number of biopsy cores; number of positive cores and the corresponding positive core ratio; maximum percentage of tumor involvement in any single core; presence of cribriform or intraductal carcinoma patterns; and perineural invasion (PNI), when present.

#### 2.2.4. Preoperative Risk Stratification Tools

For descriptive, comparative, and exploratory purposes, several established preoperative PCa risk stratification systems were calculated for all patients. These instruments were selected because they reflect routine clinical decision-making and integrate multiple clinical and pathological parameters relevant to PCa aggressiveness, namely:

- *D’Amico Risk Classification*—was assigned according to the original criteria, based on the combination of pre-biopsy PSA level, biopsy Gleason score, and cT stage [39]. Patients were categorized as: (1) Low risk: PSA < 10 ng/mL, Gleason score ≤ 6, and clinical stage cT1–cT2a. (2) Intermediate risk: PSA 10–20 ng/mL, Gleason score 7, or clinical stage cT2b. (3) High risk: PSA > 20 ng/mL, Gleason score 8–10, or clinical stage ≥ cT2c.
- *Cambridge Prognostic Group (CPG) Classification*—was calculated using preoperative PSA, biopsy ISUP GG, and cT stage, assigning patients to one of five prognostic categories (CPG1–CPG5) [42]. Compared to the three-tier D’Amico classification, CPG provides more granular stratification of localized PCa and has demonstrated improved discrimination of long-term oncological outcomes in contemporary cohorts.
- *National Comprehensive Cancer Network (NCCN) Risk Categories*—risk stratification according to NCCN guidelines was also performed for all patients, classifying cases into low, favorable intermediate, unfavorable intermediate, high, or very high-risk categories [40]. NCCN risk grouping integrates pre-biopsy PSA level, biopsy ISUP GG, cT stage, and the extent of biopsy involvement, and is widely applied in routine clinical practice to guide treatment recommendations and patient counseling.
- *The University of California, San Francisco–Cancer of the Prostate Risk Assessment (UCSF-CAPRA) score*—was calculated for each patient as a composite numerical score ranging from 0 to 10, which integrates age at diagnosis, preoperative PSA level, biopsy Gleason score/ISUP GG, cT stage, and the percentage of positive biopsy cores to stratify patients into low (0–2), intermediate (3–5), or high (6–10) risk categories [41]. This risk stratification has been validated for predicting adverse oncological outcomes following definitive treatment/RP, including biochemical recurrence, metastatic progression, and PCa-specific mortality, and was used in the present study to contextualize cumulative preoperative disease risk.

Collectively, these predictive instruments were used to contextualize baseline disease severity, reflect cumulative preoperative risk, and explore associations between traditional clinical risk stratification and ISUP GG migration. As composite prognostic tools, they were applied descriptively and in exploratory univariable analyses to assess their relationship with ISUP grade migration and CSU in PCa. However, they were not entered simultaneously with their individual component variables (e.g., PSA, PSAD, clinical stage, biopsy grade, or positive core ratio) in multivariable regression or MLMs.

This strategy was adopted to avoid multicollinearity and structural circularity, as these composite scores inherently encode information already represented by their constituent predictors. Including both a risk score and its underlying variables in the same model would artificially inflate apparent predictive performance and compromise interpretability. Accordingly, multivariable and MLMs were constructed using either individual clinical and pathological features or, in sensitivity analyses, composite risk scores alone, but not both concurrently.

#### 2.2.5. Radical Prostatectomy and Final Pathological Assessment

All patients underwent open retropubic RP, performed by experienced urologic surgeons as part of routine clinical care. Pelvic lymph node dissection was performed selectively based on preoperative risk assessment. Prostatectomy specimens were processed using whole-mount step sectioning at 3–4 mm intervals, with complete embedding. Apex and base were sectioned parasagittally. Final pathological assessment documented pathological T stage (pT2–pT3); primary and secondary Gleason patterns; final ISUP GG; surgical margin status; presence of ECE, SVI, lymphovascular invasion (LVI), and PNI; as well as number of lymph nodes examined and number of positive nodes when applicable.

### 2.3. Statistical Analysis

The primary outcome was CSU, defined as a transition from biopsy ISUP GG ≤ 2 to RP ISUP GG ≥ 3. Predictive modeling was restricted a priori to patients with biopsy ISUP GG 1–2, as this subgroup represents the population in which occult higher-grade disease has the greatest impact on treatment decision-making.

#### 2.3.1. Descriptive and Univariable Analyses

Continuous variables were assessed for normality using Shapiro–Wilk tests and Q–Q plots. Normally distributed variables were summarized as mean ± standard deviation (SD), whereas non-normally distributed variables were reported as median with interquartile range (IQR). Categorical variables were expressed as frequencies/percentages.

Univariable associations between candidate predictors and CSU were assessed using logistic regression with Firth penalization to reduce small-sample bias. Variables achieving *p* < 0.10 and those deemed clinically relevant were considered for multivariable modeling.

#### 2.3.2. Multivariable Modeling Strategy

Multivariable modeling aimed to identify independent predictors of CSU while minimizing structural and statistical bias. Biopsy ISUP grade group was not included as a predictor, given the deterministic limitation that higher grades cannot be upgraded.

Feature selection was performed using LASSO-penalized logistic regression, followed by refitting of selected predictors using Firth penalized logistic regression to obtain the final locked reference model. To assess the stability of LASSO-based feature selection under sampling perturbation, a bootstrap stability analysis was conducted. LASSO-penalized logistic regression with internal cross-validation was refit on 500 bootstrap resamples of the restricted cohort, and the selection frequency of each candidate predictor was recorded. This approach quantifies whether retained predictors are robust to small changes in the dataset or reflect idiosyncratic, sample-specific associations—a particular concern given the low event count (n = 10) and the potential for quasi-separation with sparse binary predictors.

Multicollinearity among candidate predictors was formally assessed using variance inflation factors (VIFs) and pairwise Pearson correlations. A VIF threshold of 5 was applied to identify potentially problematic collinearity.

Model discrimination was assessed using the area under the receiver operating characteristic curve (AUC). Calibration was evaluated using calibration intercept, calibration slope, and visual calibration plots. Internal validation was performed using resampling techniques to estimate optimism-corrected performance metrics.

#### 2.3.3. Machine Learning Models

Multiple ML algorithms were implemented using the same predefined feature set, including: regularized logistic regression, random forest, gradient boosting/extreme gradient boosting (XGBoost), support vector machine (SVM), and multilayer perceptron (MLP).

Prior to MLM training, continuous predictors were standardized to zero mean and unit variance (z-score normalization) to ensure comparability across features with different scales, as required by distance-based and gradient-sensitive algorithms (e.g., SVM, MLP). Categorical predictors (DRE, MRI ECE, MRI adenopathy, variant histology) were encoded as binary (0/1) variables. No feature engineering beyond the predefined clinical variables was performed. Hyperparameter tuning for each ML algorithm was conducted via nested cross-validation within the training folds to prevent information leakage. The same predefined feature set was used across all models to ensure a fair comparison.

Models were trained and evaluated using stratified cross-validation. All MLMs were benchmarked against the locked multivariable logistic regression model. No external validation cohort was available. Temporal validation by acquisition period was not performed because individual procedure dates were not retained in the de-identified analytical dataset; only the biopsy-to-RP interval was recorded. Internal validation therefore relied on repeated stratified cross-validation (5-fold, 10 repeats) and bootstrap resampling to estimate optimism-corrected performance, recognizing that these approaches cannot fully substitute for temporal or external validation. Model interpretability was assessed using: SHapley Additive exPlanations (SHAP) for global and feature-level importance, and permutation importance as a model-agnostic validation of feature contribution.

Clinical utility was evaluated using decision curve analysis (DCA) and net benefit estimation across clinically relevant threshold probabilities. All analyses were performed using Python (version 3.14.2; Python Software Foundation, Wilmington, DE, USA), employing scikit-learn (version 1.8.0), SHAP (version 0.50.0), XGBoost (version 3.1.3), NumPy (version 2.4.1), and pandas (version 2.3.3). Statistical significance was defined as a two-sided *p*-value < 0.05 throughout.

## 3. Results

### 3.1. Baseline Clinical, Imaging, and Biopsy Characteristics

The study cohort comprised 96 consecutive patients with histologically confirmed PCa who underwent pre-biopsy mpMRI, systematic ± targeted TRUS biopsy, and subsequent RP. The mean patient age at diagnosis was 66.6 ± 6.3 years (range 47–79 years). Median pre-biopsy PSA level was 9.0 ng/mL (IQR 6.3–13.8 ng/mL), with a median prostate volume of 39.5 mL (IQR 33.5–50.5), resulting in a median PSAD of 0.23 ng/mL^2^ (IQR 0.16–0.34).

DRE was suspicious for malignancy in 77 patients (80.2%), reflecting the predominantly intermediate- to high-risk profile of the cohort. All patients underwent pre-biopsy mpMRI. The index lesion was scored PI-RADS 5 in 34 cases (35.4%), PI-RADS 4 in 37 cases (38.5%), PI-RADS 3 in 21 cases (21.9%), and PI-RADS 2 in 4 cases (4.2%). Most patients harbored a single MRI-visible lesion (76 patients, 79.2%), while 20 patients (20.8%) demonstrated multifocal disease with up to four lesions identified. MRI lesions were unilateral in 65 patients (67.7%) and bilateral in 31 patients (32.3%). The mean maximal lesion diameter was 1.77 ± 0.77 cm (median 1.62 cm).

Radiologic signs of locoregional tumor extension were relatively uncommon. MRI-suspected ECE (cT3a) was reported in 12 patients (12.5%), and SVI (cT3b) in 4 patients (4.2%). Suspicious pelvic lymphadenopathy (cN1) was identified in 13 patients (13.5%). Based on clinical staging, the majority of tumors were classified as cT2 (80 patients, 83.3%), including cT2a in 51 patients (53.1%), cT2b in 4 (4.2%), and cT2c in 25 (26.0%). Twelve patients (12.5%) were staged as cT3, while four patients (4.2%) had non-palpable, incidentally detected, cT1c disease.

On systematic ± targeted biopsy, the distribution of initial ISUP GGs was as follows: GG1 in 16 patients (16.7%), GG2 in 48 patients (50.0%), GG3 in 19 patients (19.8%), GG4 in 9 patients (9.4%), and GG5 in 4 patients (4.2%). Thus, nearly 70% of the cohort presented with ISUP GG ≥ 2 disease on biopsy. According to D’Amico risk stratification, 11 patients (11.5%) were classified as low risk, 48 (50.0%) as intermediate risk, and 37 (38.5%) as high risk. The CPG classification distributed patients across all five categories, with 12 patients (12.5%) in CPG1, 32 (33.3%) in CPG2, 28 (29.2%) in CPG3, 16 (16.7%) in CPG4, and 8 (8.3%) in CPG5. Using NCCN criteria, 14 patients (14.6%) were low risk, 57 (59.4%) intermediate risk (30 favorable and 27 unfavorable intermediate), and 25 (26.0%) high or very high risk. The median UCSF-CAPRA score was 4 (IQR 3–6), corresponding to low-, intermediate-, and high-risk categories in 19 (19.8%), 52 (54.2%), and 25 patients (26.0%), respectively.

Biopsy sampling characteristics are summarized in Table 1. A median of 12 biopsy cores (IQR 12–13) were obtained per patient, with a median of 5 cancer-positive cores (IQR 2–8). The median proportion of positive cores was 44.5% (IQR 16.7–66.7%). The maximum tumor involvement in any single core reached a median of 50.5% (IQR 30–90%), and 26 patients (27.1%) had at least one biopsy core with ≥90% cancer involvement. Variant histologic patterns, including intraductal carcinoma, cribriform architecture, or other non-acinar features, were identified in 26 cases (27.1%) on final pathology.

The median interval from biopsy to RP was 111.5 days (~3.7 months). All patients underwent open retropubic RP. Pelvic lymph node dissection was performed in 36 patients (37.5%) based on preoperative risk assessment. The median number of lymph nodes removed was 10 (range 2–18). Pathologic nodal status was pN1 in 13 patients (13.5%), pN0 in 23 patients (24.0%), and pNx in 60 patients (62.5%).

### 3.2. ISUP Grade Group Concordance and Migration Between Biopsy and Prostatectomy

**Figure 1:**
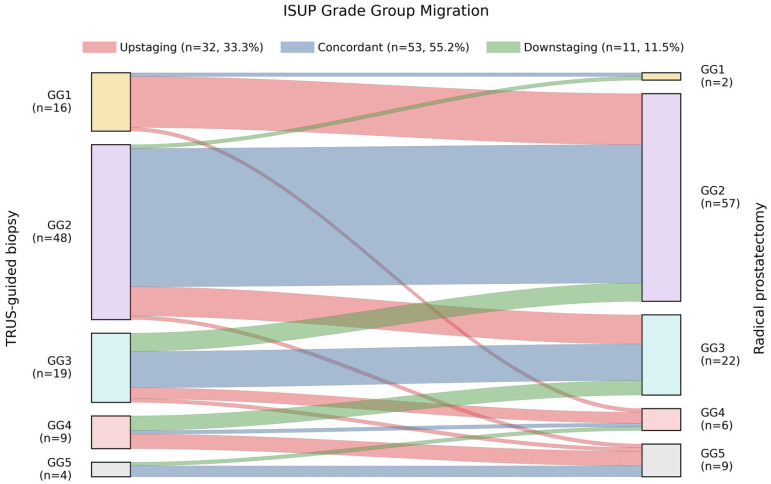
**ISUP grade group migration between biopsy and radical prostatectomy.** Sankey diagram illustrating transitions between ISUP grade groups at biopsy (left) and radical prostatectomy (right) in the full cohort (n = 96). Flow widths are proportional to patient counts. Concordant grading is shown in blue, upgrading in red, and downgrading in green. The diagram highlights the high frequency of upgrading among biopsy ISUP Grade Group 1 cases and the bidirectional instability observed in intermediate and high-grade groups.

Final RP pathology revealed a shift toward higher-grade disease compared with biopsy findings. The postoperative ISUP GG distribution was: GG1 in 2 patients (2.1%), GG2 in 57 patients (59.4%), GG3 in 22 patients (22.9%), GG4 in 6 patients (6.3%), and GG5 in 9 patients (9.4%). When biopsy and prostatectomy grades were compared, 53 patients (55.2%) demonstrated grade concordance, while 43 patients (44.8%) exhibited ISUP grade migration. Upgrading to a higher ISUP GG at RP occurred in 32 patients (33.3%), whereas downgrading occurred in 11 patients (11.5%), indicating that upgrading was approximately three times more frequent than downgrading in this MRI-informed cohort. The detailed pattern of grade transitions is illustrated in Figure 1.

Grade migration was strongly dependent on the initial biopsy ISUP GG. Among patients diagnosed with GG1 on biopsy (n = 16), 15 cases (93.8%) were upgraded at RP. Fourteen of these patients were upgraded to GG2, while one patient demonstrated a marked upgrade to GG4. Only one GG1 biopsy case remained GG1 on final pathology. Patients with GG2 on biopsy (n = 48) showed the highest concordance rate: 38 patients (79.2%) remained GG2 at RP. Nevertheless, 9 GG2 cases (18.8%) were upgraded—eight to GG3 and one directly to GG5—while a single case (2.1%) was downgraded to GG1.

Biopsy GG3 cases (n = 19) displayed more heterogeneous behavior. Ten patients (52.6%) remained GG3, three patients (15.8%) were upgraded (two to GG4 and one to GG5), and five patients (26.3%) were downgraded to GG2. In contrast, biopsy GG4 tumors (n = 9) exhibited pronounced bidirectional discordance. Only one patient (11.1%) remained GG4 at RP, whereas four patients (44.4%) were upgraded to GG5 and four (44.4%) were downgraded to GG3. Among the four patients with GG5 on biopsy, three were confirmed as GG5 at RP, while one patient was downgraded to GG4; as expected, no further upgrading was possible beyond GG5.

Overall, despite universal pre-biopsy mpMRI and combined systematic plus targeted sampling, nearly half of patients experienced ISUP grade discordance between biopsy and RP. Clinically meaningful upgrading was observed in a substantial proportion of patients initially classified as favorable or intermediate risk. Specifically, 12 patients (12.5%) were reclassified from biopsy ISUP ≤ 2 to final ISUP ≥ 3 disease, a transition with direct implications for treatment intensity and prognosis. Conversely, downgrading from biopsy ISUP ≥ 3 to final ISUP ≤ 2 occurred in only 6 patients (6.3%).

Exploratory analyses suggested that upstaged tumors were associated with more adverse pathological features at RP. Patients who experienced upgrading were more likely to harbor extra-prostatic extension (≥pT3a) and positive surgical margins compared with patients without upgrading (62.5% vs. 28.8% for ≥pT3 disease and 38% vs. 22% for positive margins, respectively), although these differences did not reach statistical significance in the present cohort. Rates of SVI, LVI, and PNI were also numerically higher in upstaged cases, consistent with their higher pathological grade and stage.

### 3.3. Analytical Workflow and Definition of the Primary Endpoint

**Figure 2:**
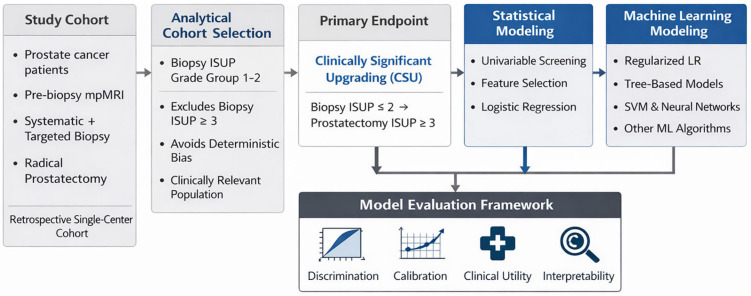
**Analytical workflow and definition of the clinically significant upgrading endpoint.** Schematic representation of the study’s analytical framework. From the full cohort of PCa patients undergoing pre-biopsy multiparametric MRI, systematic ± targeted biopsy, and radical prostatectomy, analyses were restricted to individuals with biopsy ISUP Grade Group 1–2 in order to avoid structural bias related to the impossibility of upgrading in higher biopsy grades. The primary endpoint was clinically significant upgrading, defined as a transition from biopsy ISUP ≤ 2 to prostatectomy ISUP ≥ 3. Parallel modeling strategies were implemented, including traditional multivariable logistic regression and multiple machine learning algorithms using a predefined clinical, imaging, and biopsy feature set. Model performance was evaluated across complementary domains of discrimination, calibration, clinical utility, and interpretability to identify robust and clinically actionable predictors of occult higher-grade disease.

The analytical strategy and modeling workflow are summarized in Figure 2. Given the clinical relevance of identifying patients at risk for harboring occult higher-grade disease, subsequent predictive analyses focused on CSU, defined as a transition from biopsy ISUP GG ≤ 2 to ISUP GG ≥ 3 at RP.

To avoid structural bias related to the impossibility of further upgrading in higher biopsy grades, predictive modeling was restricted to patients with biopsy ISUP GG 1–2 (n = 64). This subgroup represents the population in which misclassification has the greatest potential impact on treatment selection and counseling.

Two complementary modeling approaches were pursued in parallel: (1) traditional statistical modeling, culminating in a parsimonious, locked multivariable logistic regression model, and (2) a range of ML algorithms designed to explore potential non-linear relationships. Model performance was assessed using discrimination, calibration, and clinical utility metrics.

Traditional clinical risk stratification systems were additionally evaluated to contextualize upgrading risk within established clinical frameworks.

### 3.4. Predictors of Clinically Significant ISUP Upgrading at Radical Prostatectomy

Factors associated with CSU were evaluated using the restricted cohort defined above (biopsy ISUP GG 1–2, n = 64). As biopsy ISUP GG is structurally constrained as a predictor of upgrading (higher grades have progressively diminishing potential for further upgrading), it was not included as a standard covariate in multivariable models. In this restricted cohort, CSU occurred in 10/64 patients (15.6%), whereas 54/64 patients (84.4%) remained ISUP ≤ 2 on final pathology.

#### 3.4.1. Univariable Predictors of Clinically Significant Upgrading

Univariable logistic regression (Firth penalized) was performed across the predefined predictor set encompassing baseline clinical variables (age, PSA, PSAD, DRE), mpMRI metrics (PI-RADS, lesion diameter, lesion multiplicity and laterality, MRI-suspected SVI, ECE and adenopathy), biopsy tumor burden (positive core ratio, maximum tumor involvement per core), and the presence of a non-acinar/variant pattern, as summarized in Table 2. The aim was to evaluate the association between these variables and the risk of CSU in ISUP.

Clinical parameters, including age and absolute serum PSA level, were not significantly associated with upgrading when analyzed individually. In contrast, PSAD demonstrated a consistent positive association trend with upgrading risk, but did not reach conventional statistical significance (*p* = 0.074). Similarly, among MRI-derived variables, higher PI-RADS category, larger maximum lesion diameter, and the presence of MRI-suspected ECE were each associated positively with an increased likelihood of upgrading. Even so, none of the MRI-derived variables were significantly associated with upgrading on univariable analysis (all *p*-values > 0.1) in this restricted cohort, likely reflecting limited event counts and the confounding influence of targeted sampling on grade ascertainment. Accordingly, lesion multiplicity and bilaterality, as well as suspected SVI and/or adenopathy on MRI, showed even weaker and inconsistent associations.

Most composite preoperative risk stratification tools—including D’Amico and CPG classifications and CAPRA score—were significantly associated with CSU risk on univariable analysis (Table 2). However, because these instruments incorporate overlapping information from PSA, grade, and clinical stage, they were not simultaneously modeled with their individual component variables in subsequent multivariable analyses.

Biopsy-related tumor burden metrics emerged as particularly informative, demonstrating the strongest associations with CSU. Specifically, a higher number of positive biopsy cores (i.e., the positive core ratio) and maximum percentage involvement in any single core were both significantly associated with CSU risk (see Table 2). The presence of variant histologic patterns on biopsy showed a trend toward higher upgrading risk but did not reach statistical significance when considered in isolation.

For completeness, a parallel analysis was conducted using any ISUP grade migration (upgrading or downgrading) as the outcome. In this context, inclusion of biopsy ISUP GG as a predictor was methodologically appropriate. Lower biopsy GGs were more prone to upgrading, while higher biopsy grades—particularly GG 4—were associated with bidirectional migration. MRI and biopsy burden variables contributed modestly to migration risk, although effect sizes were attenuated compared with upgrading-specific models.

Collectively, these analyses demonstrate that ISUP upgrading is not random, even in an MRI-informed biopsy setting. Instead, upgrading is independently associated with a constellation of tumor burden metrics, PSAD, and MRI-derived features of lesion aggressiveness and extent. These findings provide a rational and biologically coherent foundation for the development of multivariable predictive models and ML approaches, which are presented in the subsequent sections.

#### 3.4.2. Multivariable Analysis of ISUP Upgrading

Multivariable modeling focused on identifying independent predictors of upgrading, while explicitly accounting for structural constraints imposed by the biopsy grade distribution. To this end, patients with biopsy GG 5 disease were excluded from upgrading models, as no further upgrading was possible in this subgroup. In fact, as previously stated, the primary outcome was CSU, therefore all the following analyses used the same predefined, clinically actionable, sub-cohort (n = 64).

A feature-based multivariable logistic regression model was constructed using variables selected from univariable screening (*p* < 0.10) and clinical relevance. In this model, after adjustment and penalized feature selection, biopsy tumor burden—captured by the positive core ratio—remained the sole independent predictor of CSU. MRI-suspected ECE also retained an independent association, suggesting that radiologic signs of local advancement capture aggressive biological behavior not fully reflected by biopsy grading alone.

In contrast, age, absolute PSA level, and lesion multiplicity did not contribute independently once PSAD and MRI features were accounted for. To address potential overfitting given the modest event count, model parsimony was prioritized. Collinearity between predictors was carefully assessed as well.

As a sensitivity analysis, an alternative multivariable model incorporating composite risk scores in place of individual component variables was evaluated. In this score-based framework, higher CAPRA score and higher CPG category were associated with upgrading, although discrimination was comparable—not superior—to the feature-based model. Given its greater interpretability and to avoid the risk of potential circularity, the feature-based model was retained as the primary multivariable analysis.

Given the limited number of CSU events (n = 10), predictor selection was performed using LASSO-penalized logistic regression to prevent overfitting and to identify the most stable predictors within the predefined feature set. LASSO retained two variables: (1) Positive core ratio and (2) PSAD.

These variables were then entered into the final multivariable model estimated using Firth penalized logistic regression, yielding the “locked model” reported in Table 3.

In the final multivariable model, positive core ratio remained independently associated with CSU. Specifically, each 10% increase in positive core ratio was associated with a 54% increase in the odds of CSU (adjusted OR 1.54, 95% CI 1.10–2.17), as detailed in Table 3. PSAD did not remain independently significant after adjustment, although its directionality remained positive.

The final locked model demonstrated good apparent discrimination for CSU (AUC 0.791). Internal validation by bootstrap resampling yielded an optimism-robust estimate of discrimination with mean AUC 0.782 (95% bootstrap percentile interval 0.684–0.811). Calibration was acceptable, with a bootstrap mean calibration slope of 1.07 (95% bootstrap percentile interval 0.30–2.07), though uncertainty was expected given the limited number of upgrading events.

Bootstrap stability analysis (500 resamples) confirmed that positive core ratio was the most frequently selected predictor (77.8%), followed by PSAD (61.6%), supporting their retention in the locked model. Notably, SVI on MRI—present in only one patient in the restricted cohort, which was also a CSU event—was selected in 45.8% of resamples despite its near-zero prevalence, consistent with quasi-perfect separation rather than a stable biological signal. All other MRI-derived and clinical variables demonstrated moderate-to-low selection frequencies (33–58%), indicating insufficient stability for independent prediction in this limited-event dataset.

Multicollinearity diagnostics confirmed that no candidate predictor exceeded a VIF of 5. Among the final model variables, the Pearson correlation between positive core ratio and PSAD was moderate (r = 0.43), with VIFs of 2.63 and 1.31, respectively, indicating that both variables contributed non-redundant information to the model. The highest observed VIF among all candidates was 2.76 (maximum tumor involvement per core), reflecting its expected collinearity with positive core ratio (pairwise r = 0.77). These values are well within acceptable limits and confirm that multicollinearity did not meaningfully distort coefficient estimates or feature selection.

However, given the possibility of nonlinear interactions between PSAD, MRI lesion characteristics, and biopsy tumor burden—particularly in small, heterogeneous cohorts—we next developed a panel of MLMs using the same restricted cohort and a pre-specified feature set, benchmarking all models against the locked multivariable reference model (Table 3).

### 3.5. Machine Learning Models for Predicting Clinically Significant Upgrading

MLMs were developed to predict CSU (biopsy ISUP ≤ 2 to RP ISUP ≥ 3) within the restricted cohort of biopsy GG 1–2 patients (n = 64). All MLMs were trained using a pre-specified feature set comprising clinical variables (age, PSAD, DRE), mpMRI variables (PI-RADS category, maximal lesion diameter, MRI-suspected ECE, MRI adenopathy), biopsy tumor burden metrics (positive core ratio, maximal tumor involvement per core), and the presence of a non-acinar/variant pattern.

Model performance was assessed using repeated stratified cross-validation (5 folds, 10 repeats), reporting discrimination (AUC), overall accuracy of probabilistic predictions (Brier score), and calibration (calibration intercept and slope). All models were benchmarked against the locked multivariable reference model based on positive core ratio and PSAD.

#### 3.5.1. Comparative Predictive Performance

Across all evaluated ML approaches, no model outperformed the locked reference model in discrimination. The locked model achieved the highest mean AUC with the most favorable calibration profile, supporting its retention as the primary predictive approach for CSU in this dataset. The full discriminative performance of the locked regression model versus all ML algorithms is summarized in Table 4.

Specifically, the locked parsimonious logistic regression model (using two variables: positive core ratio and PSAD) achieved a cross-validated mean AUC of about 0.756 (with a very small standard deviation, ~0.010) in the restricted cohort. This indicates the model’s discriminative performance was quite high in predicting CSU (biopsy ISUP ≤ 2 to RP ISUP ≥ 3) for patients with biopsy GG 1–2. Internal validation via bootstrap yielded a similar optimism-corrected AUC (~0.782) for the locked model, confirming its strong performance. While several MLMs achieved moderate discrimination, increased model complexity did not translate into superior discrimination in this dataset. However, these comparisons must be interpreted in the context of the very low effective events per variable (EPV) ratio in this cohort (as discussed further in the Discussion).

**Figure 3:**
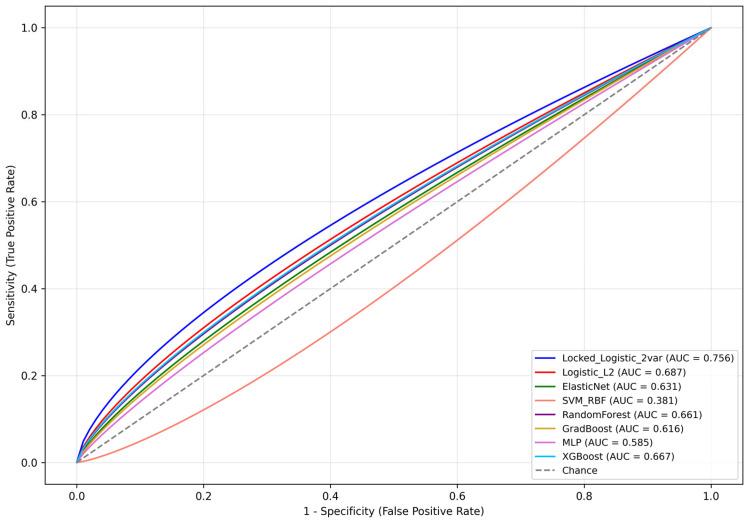
**Discrimination of predictive models for clinically significant upgrading.** Cross-validated receiver operating characteristic (ROC) curves for eight models predicting upgrading from biopsy ISUP Grade Group ≤ 2 to Grade Group ≥ 3 at radical prostatectomy (n = 64). Models shown include: (1) Locked logistic regression (2 variables)—AUC = 0.756; (2) L2-regularized logistic regression—AUC = 0.687; (3) Elastic net regression—AUC = 0.631; (4) Support vector machine with radial basis function kernel (SVM_RBF)—AUC = 0.381; (5) Random forest—AUC = 0.661; (6) Gradient boosting (GradBoost)—AUC = 0.616; (7) Multilayer perceptron (MLP)—AUC = 0.585; and Extreme gradient boosting (XGBoost)—AUC = 0.667. The locked logistic model outperformed all machine learning approaches. The dashed line indicates no-discrimination performance.

As visualized in Figure 3, among all models evaluated, the locked logistic regression model (Locked_Logistic_2var) achieved the highest discrimination (AUC = 0.756) and demonstrated superior calibration and clinical utility. Extreme gradient boosting (XGBoost) was the best-performing MLM (AUC = 0.667), achieving comparable discrimination and second-highest net benefit across thresholds, particularly at 15–20%. Random forest (AUC = 0.661) also performed competitively in terms of clinical utility. In contrast, the support vector machine with radial basis function kernel (SVM_RBF) performed poorly (AUC = 0.381), likely due to limited sample size, data sparsity, and nonlinear feature interaction miscalibration. Despite their flexibility, more complex MLMs did not surpass the locked regression model, supporting its use as a transparent and clinically interpretable benchmark for upgrading prediction in this setting.

**Figure 4:**
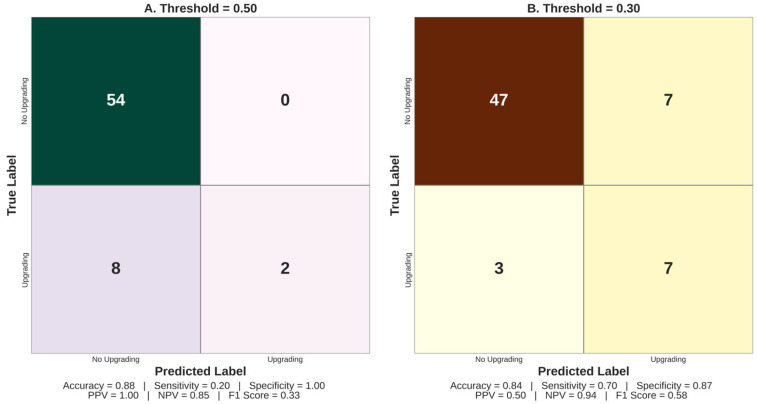
**Comparison of classification performance at two decision thresholds**: (**A**) Confusion matrix for the locked logistic regression model at the standard 0.5 threshold. (**B**) Confusion matrix at a lower 0.3 threshold, improving sensitivity but increasing false positives. Metrics include accuracy, sensitivity, specificity, positive predictive value (PPV), negative predictive value (NPV), and F1 score.

Classification performance of the locked logistic regression model was further evaluated using confusion matrices at two decision thresholds (Figure 4). At the default 0.50 threshold (Figure 4A), the model achieved excellent specificity (100%) and positive predictive value (PPV = 1.00), but low sensitivity (20%), correctly identifying only 2 of 10 upgraded patients. This conservative threshold minimizes false positives but risks underdiagnosis. By contrast, lowering the threshold to 0.30 (Figure 4B) substantially improved sensitivity (70%) and F1 score (0.58), while maintaining acceptable specificity (87%) and overall accuracy (84%). Overall, a lower threshold improved sensitivity at the expense of specificity, thus better aligning with the clinical need to minimize undertreatment, yet potentially providing more false positives. These findings underscore the impact of threshold selection on model performance and support its potential adaptation to clinical risk tolerance.

To systematically evaluate threshold sensitivity, classification metrics were computed across probability cutoffs from 0.10 to 0.50 using the locked model’s predicted probabilities (Table 5). At lower thresholds (0.10–0.15), sensitivity reached 80% with NPV ≥ 0.94, minimizing missed upgrades but at the cost of reduced specificity (63–65%) and a higher number of false-positive referrals (27–28 patients flagged). Intermediate thresholds (0.25–0.30) provided a more balanced trade-off, with sensitivity of 50–60%, specificity of 85–89%, and the highest F1 scores (0.48–0.50). Higher thresholds (≥0.40) achieved perfect specificity but missed 70% of true upgrades. The optimal threshold depends on the clinical context: in settings where undertreatment carries the greatest risk (e.g., active surveillance eligibility), a lower threshold (~0.15–0.20) may be preferred to maximize detection, whereas in settings where minimizing unnecessary intervention is prioritized, a higher threshold (~0.30–0.35) may be more appropriate. In practice, threshold selection should be individualized through shared decision-making, incorporating the patient’s values, risk tolerance, comorbidity profile, and life expectancy alongside the model’s predicted probability. Importantly, the locked model maintained positive net benefit across all evaluated thresholds relative to treat-all and treat-none strategies, confirming that threshold selection, while consequential for sensitivity–specificity balance, does not invalidate the model’s overall clinical utility.

Despite the flexibility and theoretical capacity of ML algorithms to capture non-linear relationships, none of the evaluated models consistently outperformed the parsimonious locked logistic regression model in discrimination or overall predictive stability. These findings suggest that, in this dataset, incremental model complexity did not translate into clinically meaningful gains, and that model interpretability and robustness may be prioritized over marginal improvements in apparent performance.

#### 3.5.2. Clinical Utility, Calibration, and Net Benefit

**Figure 5:**
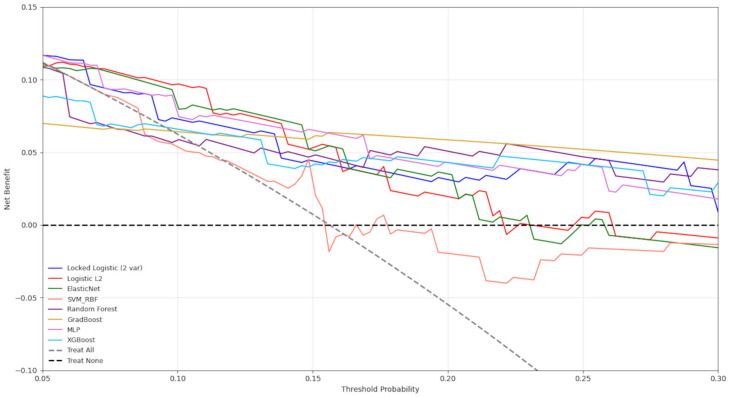
**Decision curve analysis (DCA) for predicting clinically significant ISUP upgrading**. DCA illustrates net benefit across a range of threshold probabilities for the locked logistic regression model and machine learning comparators in patients with biopsy ISUP Grade Groups 1–2 (n = 64). The locked model demonstrated consistently positive net benefit relative to treat-all and treat-none strategies across low to intermediate threshold probabilities, corresponding to clinically relevant decision ranges. Machine learning models provide limited or inconsistent incremental benefit, with XGBoost and random forest showing modest advantage only at higher thresholds. These findings support the locked model as the most clinically useful and robust approach for preoperative risk stratification.

Given that discrimination alone does not fully characterize clinical usefulness, we next evaluated model performance in terms of clinical utility and risk calibration using DCA and probabilistic calibration assessment. DCA was applied to all models using cross-validated predictions (Figure 5). The locked logistic model demonstrated the highest net benefit at lower risk thresholds (~10–15%), a range that is clinically relevant for decisions aimed at minimizing the risk of undertreatment, consistent with its conservative behavior. In practical terms, this threshold range corresponds to scenarios in which clinicians may favor intensified counseling, closer surveillance, or modification of surgical strategy despite otherwise favorable biopsy findings.

XGBoost and random forest demonstrated comparable or slightly higher net benefit at intermediate thresholds (~20–25%), whereas lower-discrimination models (SVM_RBF, MLP, ElasticNet) offered minimal or no clinical utility across the evaluated threshold range. Notably, these latter models frequently overlapped with or fell below treat-all and treat-none strategies, underscoring their limited applicability in this setting. These results confirm the clinical value of the locked model and support its use as the primary benchmark for machine learning comparators.

**Figure 6:**
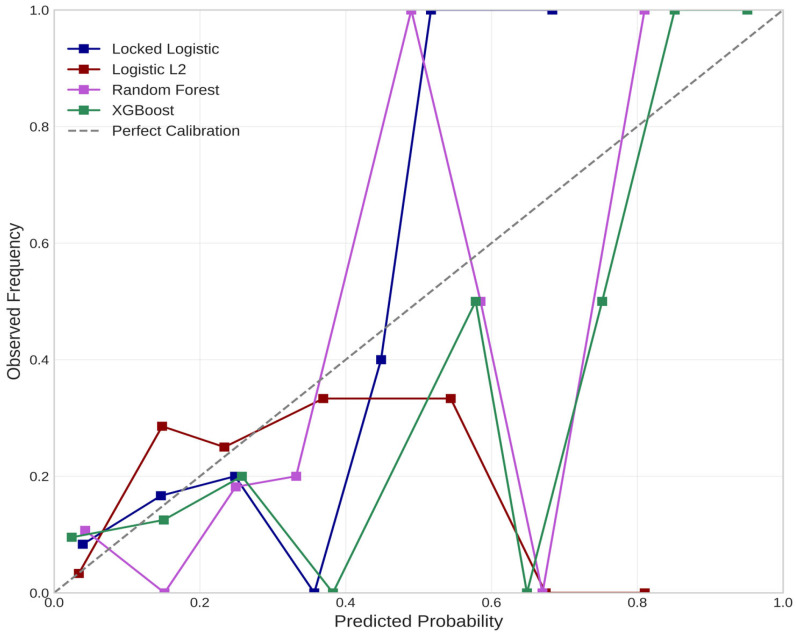
**Calibration curves for selected models predicting clinically significant upgrading.** Cross-validated calibration plots comparing predicted and observed upgrading probabilities across 10 bins. Models include: locked logistic regression, L2-regularized logistic regression (Logistic L2), random forest, and XGBoost. The locked model and XGBoost demonstrated the most consistent alignment between predicted and observed risk, particularly in low-to-intermediate risk strata. Random forest and Logistic L2 models showed inconsistent calibration, with over- and under-estimation of risk in different regions. Dashed line represents perfect calibration.

We further assessed the probabilistic calibration of selected models using decile-based calibration curves (Figure 6). The locked logistic regression model demonstrated acceptable calibration across the low- to intermediate-risk probability range, with predicted risk aligning closely with observed upgrading rates up to ~0.40–0.45. However, as expected in this low-event dataset, greater uncertainty and upward deviation were observed in higher-probability bins, reflecting sparse sampling rather than systematic model miscalibration.

Among the evaluated MLMs, XGBoost exhibited the most consistent probability dispersion across the full risk spectrum, capturing both low- and higher-risk strata with acceptable agreement between predicted and observed risk. In contrast, random forest and L2-regularized logistic regression models exhibited poorer calibration, particularly in higher-risk bins, with either systematic underestimation or flattened probability profiles, limiting their interpretability for individualized risk estimation.

Collectively, these findings indicate that the locked logistic regression model not only achieves superior discrimination but also provides clinically interpretable and reasonably calibrated risk estimates within the probability range most relevant to preoperative decision-making. This balance between discrimination, calibration, and net benefit supports its potential use as a pragmatic risk stratification tool in patients with biopsy ISUP GG 1–2 disease, where the consequences of occult upgrading are greatest.

#### 3.5.3. Model Interpretability: SHAP and Permutation Importance

To enhance interpretability and to elucidate the relative contribution of individual predictors to model behavior, we applied both SHAP and permutation importance analyses. These complementary approaches provide global and feature-level insight into the mechanisms underlying prediction of CSU.

**Figure 7:**
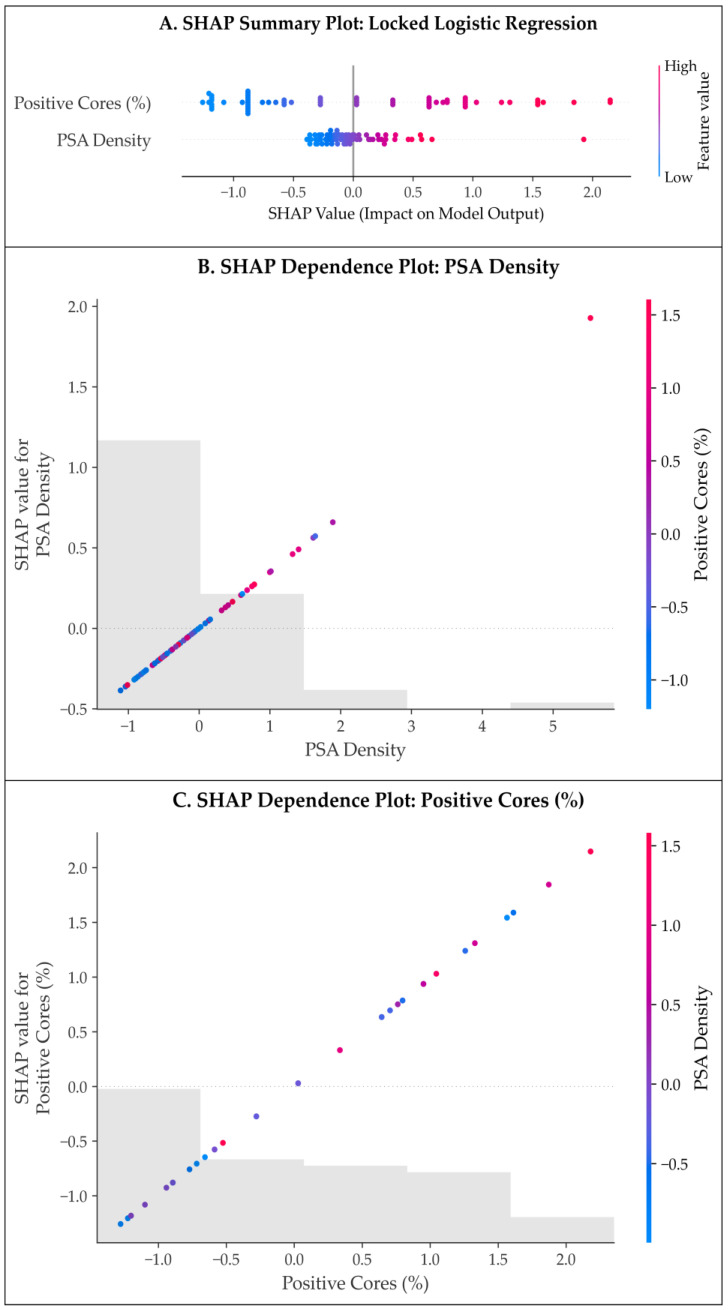
**SHapley Additive exPlanations (SHAP)-based interpretability of the locked logistic regression model:** (**A**) SHAP summary plot showing the global importance of PSA density and positive core ratio in predicting clinically significant upgrading. Each dot represents an individual patient, with color indicating the feature value and horizontal spread reflecting SHAP impact on model output; (**B**) SHAP dependence plot for PSA density, illustrating the marginal relationship between PSA density and predicted upgrading risk. The color scale represents the interacting feature (positive core ratio); (**C**) SHAP dependence plot for positive core ratio, highlighting a near-linear association with predicted upgrading probability. The color scale reflects PSA density, showing no strong interaction effect. N.B.: In all panels, the gray vertical line (**A**) and gray dashed horizontal lines (**B**,**C**) indicate a SHAP value of zero (no effect on model output). Gray bars along the x-axis in panels (**B**,**C**) represent the marginal distribution of feature values.

Global SHAP analysis of the locked logistic regression model is presented in Figure 7A. Across all patients in the restricted cohort, positive core ratio emerged as the most influential predictor of CSU, followed by PSAD. The magnitude and consistency of SHAP values indicate that these two variables dominate model output, whereas other clinical and imaging features contribute comparatively little independent information.

SHAP dependence plots further characterize the functional form of these associations. As illustrated in Figure 7B, PSAD demonstrates a non-linear relationship with predicted upgrading risk, with a steeper increase in SHAP values beyond moderate PSAD levels, consistent with a threshold-like effect. In contrast, the dependence plot for positive core ratio (Figure 7C) reveals a near-linear increase in predicted risk across its observed range, supporting its role as a robust and monotonic indicator of occult higher-grade disease. Notably, color stratification in both plots suggests minimal interaction between PSAD and positive core ratio, indicating largely additive effects within the locked model.

**Figure 8:**
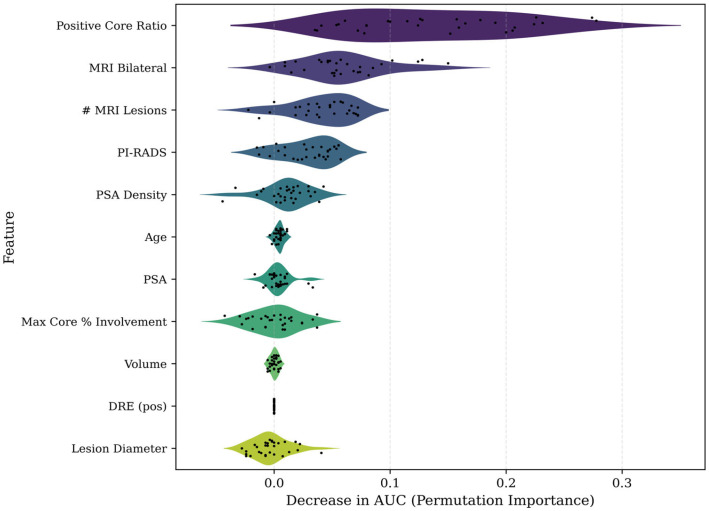
**Permutation importance for the full-feature logistic regression model.** Each violin represents the distribution of AUC drops across 30 permutation repeats for a given feature. Black dots indicate individual permutations. Greater and more consistent decreases in AUC signify stronger model dependence on that feature. Positive core ratio and PSA density consistently produced the largest drops in AUC, confirming their dominant role in model performance. N.B.: Violin colors reflect a gradient from dark purple (highest importance) to yellow-green (lowest importance), corresponding to feature ranking by median AUC decrease. Gray dashed vertical lines indicate reference gridlines at 0.0, 0.1, 0.2, and 0.3 AUC decrease. The “#” symbol denotes “number of”.

To provide a complementary, model-agnostic assessment of feature contribution, while validating the relative influence of candidate predictors, we performed permutation importance analysis on the full-feature logistic regression model (Figure 8). This method quantified the mean decrease in AUC when each feature was randomly permuted across repeated cross-validation folds.

Positive core ratio emerged as the most critical contributor to discriminative performance, followed closely by PSAD. Both variables showed consistent AUC declines across all permutations, confirming their central role in upgrading prediction. In contrast, other features such as age, lesion diameter, and prostate volume demonstrated weaker and more variable effects, suggesting they contributed less independently once core tumor metrics were accounted for. The violin distribution format further highlights the robustness of signal strength across repetitions, emphasizing the stability of the top predictors.

In summary, the locked logistic regression model based on positive core ratio and PSAD demonstrated the most favorable balance of discrimination, calibration, and clinical utility among all evaluated approaches, providing the basis for the clinical interpretation and contextualization presented in the following sections.

## 4. Discussion

In this retrospective cohort, biopsy tumor burden, particularly the positive core ratio, emerged as the dominant independent predictor, while PSAD showed a consistent but non-independent association with CSU of ISUP GG (biopsy GG ≤ 2 to RP GG ≥ 3). These findings align with the latest literature (e.g., a recent comprehensive review identified increasing percentage of biopsy core involvement and PSAD as frequent risk factors for Gleason grade upgrading [47]). PSAD has consistently emerged as a strong preoperative predictor of GG misclassification, with studies suggesting cutoffs in the 0.15–0.25 ng/mL^2^ range [47,48]. Our results reinforce this association, although PSAD did not retain independent significance after adjustment for biopsy tumor burden in the present cohort.

Notably, the observed ISUP upgrading rate in our cohort was 32%, which is in line with contemporary series reporting that ~20–40% of men still experience GG discordance at surgery despite modern diagnostic advances. A recent meta-analysis found that even with mpMRI-targeted biopsies, about 27% of cases were upgraded at RP (versus ~42% with systematic biopsy alone) [49]. This underscores that, while mpMRI and targeted biopsy have improved detection of significant PCa, a substantial subset of patients continues to have more aggressive disease on final pathology than initially indicated.

Our mpMRI-related variables (e.g., PI-RADS score) did not remain independent predictors of ISUP upgrading once biopsy tumor burden was accounted for. This reinforces the role of mpMRI primarily as a diagnostic and sampling tool rather than a standalone grading predictor. In our cohort, the patients who underwent additional mpMRI-targeted cognitive fusion lesion sampling had a nonsignificant trend toward lower upgrading rates (34.6% vs. 41.1% with systematic biopsy alone, Δ6.5%), consistent with the modest reductions in under-grading risk reported with MRI-targeted approaches [50]. Although this study may have been underpowered to detect a significant MRI effect, the direction of the trend aligns with the literature and highlights that sampling technique influences grading accuracy.

However, the marginal independent contribution of imaging variables in our multivariable model should be interpreted with caution, as several methodological factors may have systematically attenuated the predictive signal from MRI-derived features. The unrecorded heterogeneity in scanner field strength (1.5T vs. 3.0T) may have introduced measurement variability that diluted MRI-based associations with upgrading risk, despite the cross-platform intent of PI-RADS scoring. Similarly, the exclusive reliance on cognitive fusion for MRI-targeted sampling—an inherently operator-dependent technique—may have introduced systematic targeting errors that could reduce the added value of MRI-identified features by failing to adequately sample the highest-grade component of a given lesion.

Collectively, these factors may have introduced a measurement attenuation bias that underestimated the true predictive contribution of mpMRI variables. The observation that imaging features did not independently predict upgrading in our cohort should therefore not be generalized as evidence that MRI is uninformative for upgrading prediction. Rather, it may reflect the limitations of our specific imaging and sampling workflow. Studies employing standardized 3T protocols, software-assisted or in-bore fusion, and quantitative radiomic features may reveal a stronger and more independent role for imaging-derived predictors. In this regard, recent advances in MRI reconstruction—including super-resolution techniques based on partial diffusion models [51]—have demonstrated the potential to enhance spatial resolution and image fidelity beyond the constraints of acquired data. Such technological improvements could improve the quantitative accuracy of MRI-derived inputs for predictive modeling, particularly when coupled with high-dimensional radiomic extraction rather than the coarse categorical descriptors (PI-RADS score, binary ECE/SVI, lesion diameter) employed in the present study.

These results have important implications for preoperative risk stratification and treatment planning. Accurate identification of patients at risk for ISUP upgrading is critical, as misclassification can directly impact management. Active surveillance protocols, for instance, typically enroll men with biopsy ISUP GG1 (or select favorable GG2) disease under the assumption of indolent pathology. However, the substantial probability of occult higher-grade cancer in some of these men calls into question the safety of surveillance without further risk assessment [48].

An important consideration is whether these findings extend to patients with biopsy GG3 disease, which also face clinically relevant decision-making uncertainty. In our cohort, 19 patients had biopsy GG3, of whom 4 (21.1%) were upgraded to GG4–5 at RP and 5 (26.3%) were downgraded to GG2. This bidirectional grade instability, combined with the small subgroup size, precluded separate predictive modeling for GG3 patients. We note, however, that the clinical implications of GG3 upgrading differ from those of GG1–2 upgrading: GG3 patients are already classified as intermediate-risk and are typically offered definitive treatment, whereas GG1–2 patients face the critical decision between active surveillance and intervention. Our restriction to GG1–2 was therefore both methodologically motivated (to avoid structural bias) and clinically targeted at the population in which upgrading most directly alters management. Future studies with larger GG3 subgroups should evaluate whether similar tumor burden metrics predict upgrading in this population.

Prior studies have shown that patients who are upgraded from low-grade to higher-grade disease at RP are more likely to exhibit adverse pathologic features, such as extra-prostatic extension and positive margins, and they face higher rates of biochemical recurrence [10]. Upgrading, even after controlling for standard preoperative risk parameters, is a strong independent predictor of recurrence [47]. In practical terms, a man initially diagnosed with GG1 or GG2 disease who has a high PSAD or extensive biopsy involvement should be counseled that there is a significant chance of more aggressive PCa being present. Our findings support a more nuanced approach to such cases: if clinical metrics like PSAD and positive core ratio are above defined thresholds, clinicians might consider additional evaluation before committing to conservative management.

Recognition of these high-risk features could prompt confirmatory testing—repeat biopsy (potentially with enhanced targeting), multiparametric imaging, or use of tissue-based genomic assays—to ensure the absence of GG ≥ 3 disease [52]. This approach is in line with expert recommendations that patients with risk factors for upgrading undergo second-tier evaluations to reduce diagnostic uncertainty [8,47]. Conversely, for patients without such risk factors, our results may reinforce confidence in the initial grading and support decisions for surveillance or less aggressive therapy.

Ultimately, incorporating PSAD and tumor burden measures into preoperative risk models can improve patient counseling. For instance, a man with GG2 (Gleason 3 + 4) on biopsy but a positive core ratio well below 50% and PSAD < 0.15 might be a good candidate for surveillance or focal therapy, whereas the same Gleason score with >50% cores positive or PSAD > 0.20 could warrant immediate definitive treatment or at least further diagnostic workup. These nuances help personalize treatment selection, aiming to avoid both overtreatment of indolent disease, as well as undertreatment of significant PCa. Moreover, accurate preoperative grading is essential for surgical planning—for example, identifying patients who may benefit from pelvic lymph node dissection or multimodal therapy. Failure to anticipate an ISUP upgrade preoperatively could mean a missed opportunity for timely adjunctive treatment (such as concomitant lymphadenectomy or earlier initiation of adjuvant therapy), which in turn might affect long-term outcomes. Thus, improving grade concordance through better risk stratification can directly translate into more appropriate treatment decision-making.

While the association between biopsy tumor burden and upgrading risk is well established in the literature, the specific contribution of the present study lies in three areas. First, we provide a direct, head-to-head comparison of a parsimonious logistic model against multiple contemporary ML algorithms within the same mpMRI-informed cohort, demonstrating that model complexity does not automatically translate into predictive gain in this clinical context. Second, unlike prior studies that have reported individual predictors in isolation, our analysis systematically evaluated the incremental value of mpMRI-derived variables over and above biopsy tumor burden metrics, finding that imaging features did not independently contribute to upgrading prediction after accounting for positive core ratio and PSAD—a finding with practical implications for preoperative risk assessment. Third, to our knowledge, this is among the first studies to integrate comprehensive model benchmarking—including discrimination, calibration, and DCA—with SHAP-based interpretability in the specific context of ISUP CSU prediction within an mpMRI-guided biopsy pathway. We acknowledge that these findings require external validation before clinical implementation, but they provide a transparent and reproducible analytical framework that can be tested and refined in larger cohorts.

Importantly, the model presented herein is intended as a preoperative decision-support tool to complement—not replace—clinical judgment. Its primary applications include: (1) informing shared decision-making regarding active surveillance eligibility in patients with biopsy GG1–2 disease, by quantifying the individualized probability of harboring occult GG ≥ 3 cancer; (2) prompting confirmatory biopsy or additional diagnostic workup (e.g., repeat targeted biopsy, genomic testing) in patients whose predicted upgrading risk exceeds a clinician- and patient-selected threshold; and (3) refining surgical planning, including decisions regarding the extent of pelvic lymph node dissection, in patients for whom undetected higher-grade disease would alter the operative approach. As with any risk prediction tool, predicted probabilities should be interpreted within the broader clinical context, incorporating patient comorbidities, life expectancy, and individual treatment preferences.

In this dataset, the conventional multivariable logistic regression model (“locked” with predetermined predictors) outperformed more complex ML algorithms (including ensemble methods) in discrimination, calibration, and DCA. This outcome likely reflects the well-documented vulnerability of flexible algorithms to overfitting in small-sample, low-event settings rather than a fundamental inadequacy of nonlinear approaches. In moderate-sized clinical datasets, model simplicity and parsimony can be advantageous precisely because complex models lack the statistical power to reliably estimate the nonlinear interactions they are designed to capture. Thus, while ML methods have the theoretical ability to capture non-linear interactions, they also risk overfitting, especially with a limited sample (of 96 patients). In our analysis, the logistic model—using just a few key variables—achieved the best generalization on internal validation, whereas more complex models did not yield tangible gains despite their flexibility. This finding is consistent with broader evidence in the prognostic modeling literature. A systematic review encompassing 145 comparisons of MLMs versus traditional logistic regression found no significant performance benefit of ML overall, at least in terms of discrimination (AUC) [53].

In many cases, simple regression-based models are just as effective as sophisticated algorithms when predicting clinical outcomes, and they have the added benefits of transparency and interpretability [53]. Our study supports this notion: a thoughtfully constructed logistic model using readily available clinical and biopsy features was not only sufficient, but more dependable in our setting than approaches like random forests or gradient boosting. We suspect that the complex models began to “learn” noise or artifact due to the small event per predictor ratio, leading to overfitting that manifested as reduced calibration and net benefit when subjected to validation. Formally, with only 10 CSU events and up to 10 candidate features, the effective EPV ratio was approximately 1:1 in our restricted cohort, well below the commonly cited minimum of 10 EPV recommended for stable multivariable modeling and far from the threshold at which ML algorithms are expected to reliably estimate complex decision boundaries. When EPV is insufficient, differences in discrimination between ML and logistic regression tend to be negligible or favor the simpler model, not because ML is intrinsically inferior, but because it cannot learn meaningful nonlinear structure from sparse events [53]. Under such conditions, the inability of MLMs to outperform the locked logistic regression model is expected rather than surprising, and the comparison should be regarded as hypothesis-generating rather than definitive.

In contrast, the logistic model focused on the strongest predictors (PSAD and positive core ratio) remained robust. Importantly, the logistic nomogram’s simplicity facilitates clinical implementation—urologists can relatively easily calculate risk based on those factors—whereas MLMs often function as “black boxes” requiring additional tools to interpret (e.g., SHAP values). Our results therefore suggest that in the specific context of limited sample size and low event counts, a parsimonious statistical model may provide more stable and clinically trustworthy predictions than complex algorithms. This should not be interpreted as evidence that nonlinear or interaction-based modeling is inherently inferior for upgrading prediction, but rather that such approaches require substantially larger datasets to demonstrate their potential advantage.

Future research with larger cohorts could reevaluate whether advanced algorithms yield incremental benefit once a richer dataset is available, but our findings advocate for caution in assuming that ML will automatically outperform traditional approaches in this domain. Accordingly, the present study should be interpreted as an exploratory, hypothesis-generating analysis rather than a definitive validation of a clinical tool. The locked logistic model represents a candidate framework whose clinical utility must be confirmed through external validation in independent, multi-institutional cohorts before any recommendation for routine clinical adoption can be made. In addition, comprehensive model evaluation is crucial; we assessed not only discrimination but also calibration and clinical utility. The locked logistic model showed excellent calibration and net benefit across relevant threshold probabilities (per DCA), which are critical for translating predictions into practice. The more complex models did not achieve as favorable calibration—a poorly calibrated model can be misleading in a clinical setting despite a high AUC. Overall, the superior performance of the simpler model in our study provides a practical message: when data are limited, focusing on high-quality predictors and maintaining interpretability is preferable to using complex algorithms that may obscure insights without improving outcomes.

These findings should also be contextualized within the rapidly evolving landscape of AI-driven predictive modeling in urological oncology. Recent comprehensive reviews have documented a marked expansion in ML applications for PCa diagnosis, risk stratification, and outcome prediction, with increasingly sophisticated architectures being applied to imaging, genomic, and clinical data [45,46]. Several studies have demonstrated that MLMs integrating quantitative radiomic features from mpMRI—including textural heterogeneity, shape descriptors, and diffusion-derived metrics—can achieve substantially higher discrimination than models relying on conventional PI-RADS scores and lesion measurements alone [50,54]. A MLM incorporating in-bore MRI biopsy features also achieved concordance indices exceeding those of biopsy-only predictors [38]. Emerging work on MRI super-resolution and advanced reconstruction techniques may further improve the fidelity of quantitative imaging inputs, potentially enhancing the predictive power of imaging-enriched models. Our study, which relied on the conventional categorical MRI descriptors described above rather than quantitative radiomic features, was therefore not positioned to capture the full predictive potential of imaging data. Future studies integrating high-dimensional radiomic signatures with biopsy tumor burden metrics in appropriately powered cohorts may reveal complementary predictive value that was not detectable in our analysis.

## 5. Limitations and Future Directions

We acknowledge several limitations of our study. First, the retrospective design is inherently subject to selection bias and confounding. Our cohort included only patients who underwent RP, which means men who remained on active surveillance or received other treatments were not represented. This could bias our findings, as the surgical cohort might be enriched for certain risk features (e.g., younger age, patient or physician preference for definitive therapy) that are not fully captured in our variables. In particular, the upgrading rate we observed and the predictors identified might differ in an unselected population of all low- and intermediate-risk patients (including those managed non-surgically). Relatedly, because we only analyzed post-RP pathology for surgically treated men, we cannot address what the upgrade rate or model performance would be in a screened population where some might go on to radiation or continue surveillance.

Second, the sample size was relatively small (n = 96 overall, n = 64 in the restricted cohort), and critically, only 10 CSU events were observed. This extremely low EPV ratio—approximately 5 EPV for the two-variable locked model—falls below the commonly recommended minimum of 10 EPV for logistic regression, raising legitimate concerns about model stability and the reliability of reported performance metrics. Although we employed multiple strategies to mitigate this limitation—including LASSO-guided feature selection with bootstrap stability assessment, Firth-penalized regression, and repeated stratified cross-validation—predictive performance estimates should be interpreted as potentially optimistic despite these safeguards. The wide confidence intervals observed in several analyses reflect this inherent uncertainty. A larger cohort with a greater number of upgrading events would be necessary to confirm the stability of the selected predictors, refine effect size estimates, and enable more robust MLM training. Indeed, the small sample and event count also constrained the complexity of any MLMs—complex models generally require large datasets to reliably learn patterns, and in their absence, performance may stagnate or degrade. With only 10 CSU events, automated feature selection is inherently sensitive to quasi-separation from rare binary predictors. Although bootstrap stability analyses supported positive core ratio as a robust signal, selection of low-prevalence MRI extension variables (e.g., SVI, present in one patient) was driven by perfect outcome separation rather than generalizable predictive value. Model interpretation should therefore be considered exploratory, and external validation is required.

Third, this was a single-center study at a tertiary institution. Surgical pathology was evaluated by our local pathologists and biopsies were largely performed by a limited number of urologists. While this helps ensure consistency in grading within the study, it may limit generalizability. Our institution’s patient demographics and clinical practices (e.g., thresholds for recommending surgery or imaging) may differ from other centers. For example, about half of our total case-load did not undergo pre-biopsy MRI due to resource and referral patterns; other centers with universal MRI might observe different upgrading dynamics. This limitation should be considered when interpreting the non-significant associations observed for imaging variables. Additionally, MRI examinations were performed on both 1.5T and 3.0T scanners, and field-strength information was not captured in our database, precluding stratified analysis or harmonization of imaging-derived variables. Furthermore, well-documented inter-reader variability in PI-RADS scoring [21]—particularly for PI-RADS 3 lesions—may have introduced additional classification noise. These methodological limitations likely contributed to the measurement attenuation bias discussed above. External validation in multi-center datasets is needed to confirm that our findings apply broadly.

Fourth, we relied on cognitive fusion for MRI-targeted biopsies rather than an automated software fusion or in-bore MRI-guided approach. Cognitive fusion (visual estimation of lesion location on ultrasound) is inherently operator-dependent—targeting errors could lead to missing the highest-grade portion of a tumor. This limitation might have contributed to some of the upgrading we observed, as undetected high-grade foci could remain in the prostate if biopsy sampling was suboptimal. Furthermore, operator-level data were not recorded herein, thus precluding formal assessment of inter-operator variability as a confounder. While the small number of experienced biopsy operators within our center likely mitigated gross inconsistencies, residual operator-dependent targeting accuracy may have influenced upgrading rates and cannot be excluded. Although combining systematic with cognitive-targeted biopsy was our standard (which should mitigate random misses to an extent), it is possible that a dedicated software-fusion platform or direct in-bore MRI biopsy could have improved lesion sampling accuracy.

MRI in-bore targeted biopsy has been shown to achieve very high concordance with whole-gland pathology by providing real-time lesion localization and sampling guidance [38]. The lack of such technology in our workflow is a limitation. That said, recent data suggest that cognitive fusion can perform comparably to software fusion in experienced hands. Hung et al. reported no significant difference in clinically significant PCa detection between the two approaches (odds ratio ~1.46 favoring cognitive, 95% CI overlapping 1) [55]. This implies that our use of cognitive targeting might not drastically lower cancer detection overall, but it remains plausible that subtle accuracy losses could affect the detection of the highest-grade elements and thus influence upgrading rates. Additionally, we did not employ any advanced biopsy techniques like transperineal mapping or saturation biopsy, which could potentially further reduce sampling error but were beyond the scope of our typical practice. Separately, the absence of retained procedure dates in our de-identified dataset precluded temporal split validation, which would have provided a stronger approximation of real-world deployment performance. Future studies should incorporate calendar-time splits to assess model stability over evolving clinical practices and imaging protocols.

Finally, we must note that the study focused only on ISUP upgrading (and we defined “CSU” as reaching GG ≥ 3). We did not delve into downgrading cases or those upgraded within the lower grade range (e.g., GG1 to GG2) in detail. While our primary interest was identifying factors for missing high-grade disease, future analyses could also consider the patterns of downgrading—for instance, whether some GG2 on biopsy are actually over-called and turn out to be GG1 at RP—as this has its own clinical implications in terms of potential overtreatment.

Building on our findings, several avenues for future research and improvement in preoperative risk assessment are evident. Firstly, external validation of our predictive model is a priority. We plan to test the logistic nomogram in external cohorts, ideally from multiple institutions and including more diverse patient populations. An external validation would assess calibration and discrimination in a new setting and ensure the model’s generalizability. If it performs well, it could be incorporated into clinical practice as a decision support tool. If it underperforms, that would indicate the need to recalibrate or augment the model with additional predictors.

Furthermore, prospective evaluation is warranted. A prospective study could enroll men with biopsy GG1–2 who meet certain criteria (e.g., intermediate PSAD or core ratios) and follow them through confirmatory testing and treatment to observe upgrading rates. This would provide higher-level evidence (avoiding some biases of retrospective design) and could clarify cause-effect relationships. For example, a prospective trial could test whether intervening on high-PSAD patients (with immediate surgery or supplemental biopsy) improves outcomes compared to standard surveillance—essentially using our risk factors to stratify management and measuring the impact.

Beyond prospective clinical validation, integration of advanced imaging analytics may further enhance predictive accuracy. As discussed above, quantitative radiomic features extracted from mpMRI have shown promise in capturing surrogates of tumor aggressiveness that are not reflected by conventional categorical descriptors. Future studies should evaluate whether combining radiomic signatures with biopsy tumor burden metrics and PSAD in a hybrid model yields superior net benefit in appropriately powered, multi-institutional cohorts.

Lastly, the inclusion of genomic classifiers and molecular biomarkers holds promise for refining risk stratification. In recent years, assays like Decipher, Oncotype DX Genomic Prostate Score, and Prolaris (which evaluate gene expression in biopsy tissue) have been shown to predict adverse pathology and outcomes independent of clinical variables. Specifically, a higher Decipher score has been associated with greater odds of grade reclassification: in a cohort of men on active surveillance, each 0.1 increase in the Decipher score raised the odds of subsequent ISUP GG upgrade by roughly 37% [52]. Incorporating such genomic data could improve our ability to discern which GG 1–2 PCa are truly low risk and which are molecularly primed to behave aggressively.

Likewise, germline genetic factors are an emerging area of interest—for example, a recent prospective study suggested that certain inherited genetic variants might predispose men with initial GG 1 to harbor higher-grade disease at RP [56]. Though preliminary, these insights hint that personalized genetic risk scores might one day augment clinical models. Beyond genomics, other biomarkers such as serum or urinary assays could be explored. Markers like the Prostate Health Index (PHI), 4Kscore, or urinary exosomal RNA signatures have been linked to tumor grade and could be studied specifically for upgrading prediction. Integrating multi-modal data—clinical, imaging, and molecular—is a frontier for improving preoperative risk discrimination.

Finally, ongoing improvements in biopsy technology should continue to be assessed. The advent of PSMA PET imaging, for instance, could potentially identify occult high-grade lesions (e.g., detecting smaller aggressive tumors via metabolic activity) which might inform targeted biopsy or immediate treatment. While PSMA PET is currently more focused on staging rather than grading, its role in initial risk stratification could grow. In summary, future efforts should aim for more personalized and accurate pre-treatment risk profiling. Key priorities include validating our findings in broader settings, leveraging novel imaging and molecular diagnostics, and testing these strategies in prospective clinical trials to determine whether they translate into improved patient outcomes.

## 6. Conclusions

In this retrospective cohort of patients undergoing mpMRI-guided prostate biopsy and RP, CSU in ISUP GG remained a frequent and therapeutically relevant phenomenon among individuals initially classified as biopsy GG 1–2. Our exploratory analysis suggests that quantitative measures of biopsy tumor burden—most notably the positive core ratio—may represent robust predictors of occult higher-grade disease in this clinical context.

While PSAD showed a consistent positive association with upgrading risk and contributed to overall model interpretability, it did not independently improve prediction once biopsy tumor burden was accounted for. Importantly, mpMRI-derived variables did not retain independent predictive value in multivariable models, underscoring that residual upgrading risk after MRI-guided biopsy is largely driven by histologic sampling extent rather than imaging features alone.

A parsimonious logistic regression model based on positive core ratio (with supportive contribution from PSAD) achieved superior discrimination, calibration, and net clinical benefit compared with multiple ML algorithms in this exploratory single-center analysis. These results, while promising, require external validation before clinical implementation can be recommended. Nonetheless, they emphasize that, in moderate-sized clinical datasets with limited event counts, model simplicity and biological coherence may yield more stable and generalizable predictions than increased algorithmic complexity, though this observation should not preclude re-evaluation of MLMs in larger, multi-institutional cohorts.

Incorporation of biopsy tumor burden metrics into preoperative risk assessment may improve patient counseling, refine treatment selection, and reduce the risk of undertreatment in men with apparently low- or intermediate-risk PCa. Future work should focus on external validation in multi-institutional cohorts—which we consider essential before any clinical implementation—prospective evaluation, and the integration of emerging imaging and molecular biomarkers to further enhance preoperative risk stratification.

## Figures and Tables

**Table 1:** Baseline Clinical, Imaging, and Biopsy Characteristics of the Study Cohort (n = 96).

Variable	Value
Age, years	66.6 ± 6.3 (range 47–79)
Pre-biopsy PSA, ng/mL	9.0 (IQR 6.3–13.8)
Prostate volume, mL	39.5 (IQR 33.5–50.5)
PSA density, ng/mL^2^	0.23 (IQR 0.16–0.34)
Positive DRE, n (%)	77 (80.2)
PI-RADS score, n (%)	
– PI-RADS 2	4 (4.2)
– PI-RADS 3	21 (21.9)
– PI-RADS 4	37 (38.5)
– PI-RADS 5	34 (35.4)
Number of MRI lesions, n (%)	
– Single lesion	76 (79.2)
– Multiple lesions (2–4)	20 (20.8)
MRI lesion laterality, n (%)	
– Unilateral	65 (67.7)
– Bilateral	31 (32.3)
Maximal lesion diameter, cm	1.77 ± 0.77 (median 1.62)
MRI extracapsular extension, n (%)	12 (12.5)
MRI seminal vesicle invasion, n (%)	4 (4.2)
Suspicious pelvic lymph nodes (cN1), n (%)	13 (13.5)
Clinical T stage, n (%)	
– cT1c	4 (4.2)
– cT2a	51 (53.1)
– cT2b	4 (4.2)
– cT2c	25 (26.0)
– cT3 (a–b)	12 (12.5)
ISUP grade group at biopsy, n (%)	
– Grade Group 1	16 (16.7)
– Grade Group 2	48 (50.0)
– Grade Group 3	19 (19.8)
– Grade Group 4	9 (9.4)
– Grade Group 5	4 (4.2)
D’Amico risk group, n (%)	
– Low	11 (11.5)
– Intermediate	48 (50.0)
– High	37 (38.5)
CPG classification, n (%)	
– CPG1	12 (12.5)
– CPG2	32 (33.3)
– CPG3	28 (29.2)
– CPG4	16 (16.7)
– CPG5	8 (8.3)
NCCN risk category, n (%)	
– Low	14 (14.6)
– Favorable intermediate	30 (31.3)
– Unfavorable intermediate	27 (28.1)
– High/very high	25 (26.0)
CAPRA score	4 (IQR 3–6)
Biopsy cores obtained	12 (IQR 12–13)
Positive biopsy cores	5 (IQR 2–8)
Positive core ratio	44.5% (IQR 16.7–66.7)
Max. tumor involvement per core, %	50.5 (IQR 30–90)
≥90% involvement in ≥1 core, n (%)	26 (27.1)
Variant histology on final pathology, n (%)	26 (27.1)
Biopsy-to-surgery interval, days	111.5 (~3.7 months)

Continuous variables are presented as mean ± standard deviation or median (interquartile range [IQR]), as appropriate. Categorical variables are presented as number (percentage). Abbreviations: PSA = prostate-specific antigen; DRE = digital rectal examination; MRI = magnetic resonance imaging; PI-RADS = Prostate Imaging Reporting and Data System; CPG = Cambridge Prognostic Group; NCCN = National Comprehensive Cancer Network; CAPRA = Cancer of the Prostate Risk Assessment score.

**Table 2:** Univariable Predictors of Clinically Significant Upgrading (Biopsy ISUP ≤ 2 → RP ISUP ≥ 3) in the Restricted Cohort (n = 64).

Predictor	Unit (Scaled)	OR (95% CI)	*p*-Value
Age	per 5 years	0.93 (0.52–1.66)	0.794
PSA	per 5 ng/mL	1.10 (0.70–1.72)	0.68
PSA density	per 0.1 ng/mL^2^	1.39 (0.97–2.00)	0.074
Digital rectal exam	positive vs. negative	2.27 (0.35–14.82)	0.393
PI-RADS score	per 1 category	0.97 (0.45–2.09)	0.946
MRI lesion multiplicity	≥2 lesions vs. 1 lesion	1.30 (0.33–5.10)	0.708
MRI lesion laterality	bilateral vs. unilateral	1.40 (0.29–6.72)	0.664
Max. lesion diameter on MRI	per 0.5 cm	1.33 (0.87–2.03)	0.194
MRI extracapsular extension	present vs. absent	1.77 (0.21–15.08)	0.601
MRI seminal vesicle invasion	present vs. absent	1.20 (0.13–11.16)	0.885
MRI adenopathy	present vs. absent	0.70 (0.02–22.89)	0.842
Variant histology pattern	present vs. absent	2.93 (0.71–12.06)	0.136
Total biopsy cores	per 1 core	0.95 (0.82–1.10)	0.505
Positive biopsy cores (count)	per 1 core	1.50 (1.10–2.05)	0.007
Positive core ratio	per 0.1 (10%)	1.55 (1.15–2.10)	0.004
Max. tumor involvement per core	per 10%	1.33 (1.04–1.71)	0.023
D’Amico risk category	per category *	1.75 (1.03–2.98)	0.038
CPG risk category	per category **	1.40 (1.05–1.88)	0.020
CAPRA score	per 1 point (0–10)	1.25 (1.01–1.55)	0.041
NCCN risk category	per category ***	1.62 (0.94–2.80)	0.080

* D’Amico risk categories were modeled as an ordinal variable across three levels (low, intermediate, high). ** CPG risk categories were modeled as an ordinal variable across five levels (CPG1–CPG5). *** NCCN risk categories were modeled as an ordinal variable across five levels (low, favorable intermediate, unfavorable intermediate, high, very high). Univariable logistic regression was estimated using Firth penalization to reduce small-sample bias. Continuous predictors were scaled to clinically interpretable units as shown. OR = odds ratio; CI = confidence interval. Statistically significant variables (*p* < 0.05) are marked in red.

**Table 3:** Final Multivariable Model for Clinically Significant Upgrading (Locked Model; n = 64).

Predictor	Scaling	Coefficient (β)	Adjusted OR (95% CI)	*p*-Value
Positive core ratio	per 0.1 (+10%)	0.432	1.54 (1.10–2.17)	0.0125
PSA density	per 0.1 ng/mL^2^	0.198	1.22 (0.78–1.92)	0.377

Final model was selected via LASSO within the predefined predictor set and refit using Firth penalized logistic regression to mitigate small-event bias. Continuous predictors were scaled as shown to yield clinically interpretable adjusted ORs. Model intercept (β_0_): −4.526 (Firth penalized).

**Table 4:** Cross-Validated Performance of the Locked Reference Model vs. Machine Learning Models (Restricted Cohort, n = 64).

Model	AUC (Mean ± SD)	Brier Score (Mean ± SD)	Calibration Intercept (Mean ± SD)	Calibration Slope (Mean ± SD)
Locked logistic (2 variables)	0.756 ± 0.010	0.107 ± 0.003	−0.359 ± 0.113	0.732 ± 0.075
Logistic regression (full feature set, L2)	0.687 ± 0.037	0.136 ± 0.008	−0.863 ± 0.149	0.405 ± 0.078
XGBoost	0.667 ± 0.041	0.133 ± 0.009	−0.572 ± 0.187	0.641 ± 0.095
Random forest	0.661 ± 0.035	0.116 ± 0.005	−0.799 ± 0.311	0.498 ± 0.193
ElasticNet	0.631 ± 0.056	0.149 ± 0.017	−1.001 ± 0.203	0.198 ± 0.069
Gradient boosting	0.616 ± 0.072	0.164 ± 0.034	−1.166 ± 0.251	0.128 ± 0.070
MLP	0.585 ± 0.064	0.152 ± 0.019	−1.103 ± 0.241	0.197 ± 0.085
SVM_RBF	0.381 ± 0.070	0.228 ± 0.015	−1.239 ± 0.211	0.093 ± 0.052

Performance estimates were obtained using repeated stratified cross-validation (5-fold, 10 repeats). Calibration intercept and slope were calculated by fitting a calibration model to cross-validated predicted probabilities. The locked model delivered the best overall balance of AUC, Brier score, and calibration, and therefore remained the benchmark. The full-feature logistic model L2 did not improve discrimination, suggesting that additional MRI variables and secondary biopsy descriptors did not add stable incremental information beyond biopsy tumor burden in this limited cohort. Tree-based models did not improve discrimination and were generally less well calibrated.

**Table 5:** Threshold Sensitivity Analysis for the Locked Logistic Regression Model Predicting Clinically Significant Upgrading (Restricted Cohort, n = 64).

Threshold	Sensitivity	Specificity	PPV	NPV	Accuracy	F1 Score	Patients Flagged
0.10	0.80	0.63	0.29	0.94	0.64	0.42	28
0.15	0.80	0.65	0.30	0.95	0.67	0.43	27
0.20	0.60	0.74	0.30	0.91	0.72	0.40	20
0.25	0.60	0.85	0.43	0.92	0.81	0.50	14
**0.30**	**0.50**	**0.89**	**0.45**	**0.91**	**0.83**	**0.48**	**11**
0.35	0.40	0.93	0.50	0.89	0.86	0.44	8
0.40	0.30	1.00	1.00	0.89	0.89	0.46	3
0.45	0.30	1.00	1.00	0.89	0.89	0.46	3
**0.50**	**0.30**	**1.00**	**1.00**	**0.89**	**0.89**	**0.46**	**3**

Classification metrics were computed using predicted probabilities from the locked Firth-penalized logistic regression model (Table 3: positive core ratio + PSA density). Bold rows indicate the two thresholds illustrated in Figure 4 (0.30 and 0.50). “Patients flagged” denotes the total number of patients classified as positive (true positives + false positives) at each threshold. PPV = positive predictive value; NPV = negative predictive value. CSU event prevalence: 10/64 (15.6%).

## Data Availability

Data available on request.

## Abbreviations

The following abbreviations are used in this manuscript:

AUC	Area under the receiver operating characteristic curve
CAPRA	Cancer of the Prostate Risk Assessment score
CPG	Cambridge Prognostic Group
CSU	Clinically significant upgrading
DCA	Decision curve analysis
DRE	Digital rectal examination
ECE	Extracapsular extension
EPV	Events per variable
GG	Grade Group
IQR	Interquartile range
ISUP	International Society of Urological Pathology
LVI	Lymphovascular invasion
ML	Machine learning
MLP	Multilayer perceptron
mpMRI	Multiparametric magnetic resonance imaging
MRI	Magnetic resonance imaging
NCCN	National Comprehensive Cancer Network
NPV	Negative predictive value
PCa	Prostate cancer
PI-RADS	Prostate Imaging Reporting and Data System
PNI	Perineural invasion
PPV	Positive predictive value
PSA	Prostate-specific antigen
PSAD	Prostate-specific antigen density
RBF	Radial basis function
ROC	Receiver operating characteristic
RP	Radical prostatectomy
SD	Standard deviation
SHAP	Shapley Additive Explanations
SVI	Seminal vesicle invasion
SVM	Support vector machine
TNM	Tumor–Node–Metastasis (staging system)
TRUS	Transrectal ultrasound
XGBoost	Extreme Gradient Boosting
